# Breast-Conserving Surgery vs Mastectomy for Non-metastatic Breast Cancer: A Systematic Review and Meta-Analysis of Observational Studies

**DOI:** 10.7759/cureus.88612

**Published:** 2025-07-23

**Authors:** Daniela Fulginiti, Sabrina S Domene, Carla Isabella Miret Durazo, Vanessa Boosahda, Thomas Campos Carmona, Karla Lora-Tavarez, Hrachya Ajamyan, Haya Al Shakkakee, Pallavi Shekhawat, Oxiris Yexalen Garcia Gonzalez

**Affiliations:** 1 General Practice, Pontifical Catholic University of Argentina, Buenos Aires, ARG; 2 General Practice, Universidad Nacional de Mar del Plata, Mar de Plata, ARG; 3 General Practice, Centro de Estudios Universitarios Xochicalco, Escuela de Medicina Tijuana, Tijuana, MEX; 4 General Practice, Xavier University School of Medicine, Oranjestad, ABW; 5 General Practice, Universidad de Ciencias Médicas, San Jose, CRI; 6 General Practice, Universidad Nacional Pedro Henríquez Ureña, Santo Domingo, DOM; 7 General Medicine, Yerevan State Medical University After Mkhitar Heratsi, Yerevan, ARM; 8 Medicine, University of Baghdad - Al Kindy College of Medicine, Baghdad, IRQ; 9 Obstetrics and Gynecology, ESI-Post Graduate Institute of Medical Sciences and Research, Delhi, IND; 10 General Practice, Monterrey Institute of Technology and Higher Education, Zapopan, MEX

**Keywords:** breast cancer, breast-conserving surgery, disease-free survival, mastectomy, overall survival

## Abstract

Breast cancer is a leading cause of cancer-related deaths among women worldwide, accounting for 15% of all cancer deaths. The decision between breast-conserving surgery (BCS) and mastectomy (MX) plays a fundamental role in the management of early-stage breast cancer. Improved survival rates are an essential outcome influencing these decisions. This systematic review and meta-analysis aim to compare the effectiveness of BCS versus MX in terms of survival and recurrence outcomes. We conducted a comprehensive search of PubMed MEDLINE, Web of Science, Cochrane, and EMBASE databases on 03/19/2024, following the Preferred Reporting Items for Systematic Reviews and Meta-Analyses (PRISMA) 2020 guidelines. Studies published from 1994 to 2024 comparing BCS with MX in breast cancer patients were included. The meta-analysis included 22 studies with a total sample size of 389,465 participants. For local recurrence, the random-effects model indicated an odds ratio (OR) of 1.65 (95% CI: 0.79-3.47, p = 0.19, I² = 70.6%). Regional recurrence showed an OR of 1.13 (95% CI: 0.26-5.03, p = 0.87, I² = 83.8%). For disease-free survival at five years, the hazard ratio (HR) was 0.78 (95% CI: 0.57-1.09, p = 0.14, I² = 98.2%), and at 10 years, the HR was 1.12 (95% CI: 0.79-1.58, p = 0.53, I² = 96.4%). Overall survival (OS) at five years showed a significant benefit for BCS (HR = 0.49, 95% CI: 0.34-0.71, p = 0.0001, I² = 98.8%), as did OS at 10 years (HR = 0.62, 95% CI: 0.42-0.91, p = 0.0149, I² = 98.8%). High heterogeneity was present in the survival outcomes (I² > 90%), limiting the robustness of the findings and suggesting variability across studies. BCS may offer comparable or superior survival outcomes compared to MX, but further research is needed to address the substantial heterogeneity and to develop personalized treatment guidelines. Notably, all 22 cohort studies were rated “good quality” on the Newcastle-Ottawa scale (NOS), supporting the overall reliability of the evidence.

## Introduction and background

Breast cancer is the leading cause of cancer-related deaths among women worldwide, accounting for 15% of all cancer deaths [1]. In 2020, it was estimated that 685,000 women died from breast cancer globally [2]. The lifetime risk of being diagnosed with breast cancer is one in eight for women [3].

For women diagnosed with primary breast cancer, the initial treatment typically involves curative breast surgery. Historically, breast cancer surgery was dominated by Halsted's mastectomy (MX), introduced in 1894, which remained standard for decades ​[4]​. In the late 20th century, two randomized trials demonstrated that breast-conserving surgery (BCS) with radiation achieved equivalent survival to MX in early-stage breast cancer ​[4]. Advances in imaging and screening have since enabled earlier detection of nonpalpable tumors, prompting more localized and tailored surgical approaches ​[4]. Modern surgical strategies increasingly emphasize personalization, considering tumor biology, breast size, and patient preference, and minimally invasive techniques aimed at preserving quality of life without compromising oncologic outcomes.

MX remains an option for many women with screen-detected, early-stage breast cancer. While chemotherapy decisions are primarily based on tumor biology and are generally independent of surgical choice, some patients may opt for MX to avoid radiation therapy and its associated side effects, provided that they meet criteria that do not require post-MX radiation ​[4]​. Additionally, it may be necessary for women who have received radiation on the affected side or for those with relatively small breasts in the case of a large primary breast cancer, extensive calcifications, or multicentric disease. Several studies have shown no detectable difference in overall survival (OS) (the length of time a patient lives after diagnosis or the start of treatment) or disease-free survival (DFS) (the period after primary treatment during which no signs of cancer recurrence or progression are observed) between women with early-stage breast cancer who undergo BCS plus radiation and those who undergo MX ​[4,5]. However, advances in systemic therapies, including targeted agents and immunotherapy, may differentially influence long-term outcomes based on tumor biology and surgical approach, underscoring the need to individualize treatment plans. A 20-year follow-up of a randomized study comparing BCS with MX for early breast cancer showed that the rates of death from all causes for BCS were 41.7% and 41.2% for MX [6]. The respective rates of death from breast cancer were 26.1% and 24.3%, showing no differences in long-term survival rates between the two treatments [6]. A 10-year, follow-up, single-center, real-world study conducted in China showed similar results, with no statistical difference in the 10-year breast cancer-specific survival rate (91.7% vs. 70.3%), as well as the 10-year DFS rate (87.6% vs. 83.8%) [7].

The choice between BCS and MX is influenced by factors such as complication rates, length of hospital stay, rehabilitation time, patient-reported symptoms, body image, and quality of life. MX is often recommended to avoid postoperative radiotherapy (RT) ​[5]​. BCS followed by RT is the standard local treatment for early-stage invasive breast cancer; however, it is not recommended for patients with a high risk of local recurrence, such as those with aggressive tumor biology, multifocal tumors, positive margins, or certain genetic mutations, where MX may be preferred ​[8].

While the equivalence of BCS and MX for survival outcomes has been well-established through randomized controlled trials (RCTs), observational studies are essential to understanding real-world outcomes, including patient quality of life, complications, and long-term satisfaction. Observational studies provide insights into how these treatments perform outside the controlled settings of clinical trials and help identify factors that influence treatment choices and outcomes in diverse populations. While RCTs have established comparable survival rates, there is limited synthesis of real-world outcomes in diverse populations from observational data.

In this review, we seek to perform an analysis that addresses variables such as local recurrence, regional recurrence, mortality, OS, and DFS. In conducting this systematic review and meta-analysis, we adhere to the Preferred Reporting Items for Systematic Reviews and Meta-Analyses (PRISMA) standards and evaluate the methodological quality of the included studies. It is important to acknowledge that the data from these studies remain observational, which may introduce certain biases. However, observational data are crucial for informing clinical practice and patient decision-making by complementing the findings of RCTs with evidence from real-world settings.

## Review

Methods

This systematic review and meta-analysis employed the PRISMA 2020 guidelines to conduct a comprehensive systematic review [9]. The protocol was registered with the International Prospective Register of Systematic Reviews (PROSPERO), in accordance with PRISMA guidelines (PROSPERO CRD 42024530103).

Criteria for Considering Studies in This Review

Types of study: We systematically reviewed relevant studies published from 1994 to 2024 to ensure the most recent and rigorous information available in English. We meticulously screened and analyzed cohort studies and case controls. We excluded case series, cross-sectional, dissertations, book chapters, protocol articles, reviews, news articles, conference abstracts, comments, letters to the editor, and editorials. Furthermore, we excluded studies that did not clearly describe their operationalizations, were duplicated, were unable to describe their operationalizations clearly, and could not obtain the necessary data or receive a response from the original author via email.

Type of participants: This study has set specific participant selection criteria, including individuals aged 18 years and older diagnosed with breast cancer without metastasis, despite sub-classification. We plan to analyze subgroups according to the classification of breast cancer. Furthermore, we included all types of molecular subtypes and histological grades. Studies were included irrespective of the study sample size, inpatient/outpatient setting, and geography if they provided data needed for the systematic review. Exclusion criteria are individuals under 18 years of age.

Types of intervention: The study has established specific participant selection criteria, including studies that use BCS as an intervention compared with MX. Studies comparing the intervention were included. Exclusion criteria consist of studies published solely as abstracts, studies that do not directly compare these two interventions, and studies without complete information.

Outcomes

The primary outcomes to be measured in this project include local recurrence, regional recurrence, mortality, OS, and disease-free survival.

Searching Strategy

We searched PubMed MEDLINE, Web of Science, Cochrane, and EMBASE using the following terms: ‘’Mastectomy‘’, ‘’Breast conserving surgery’’, and ‘’Breast cancer'' on 03/19/2024 (Tables 1-4). This rigorous approach resulted in a homogeneous dataset, allowing for more accurate and reliable results.

**Table 1:** PubMed search

Search	Results
("Breast Neoplasms"[Mesh] OR "breast cancer"[Title/Abstract]) AND ("Mastectomy"[Mesh] OR "mastectomy"[Title/Abstract]) AND ("Breast Conserving Surgery"[Mesh] OR "conservative breast surgery"[Title/Abstract] OR "lumpectomy"[Title/Abstract])	2823

**Table 2:** Web of Science search

ID	Search	Result
1	ALL=breast cancer	757,559
2	ALL=mastectomy	34,101
3	ALL=breast conserving surgery	14,276
4	ALL=conservative breast surgery	4,978
5	ALL=lumpectomy	6,555
6	#3 OR #4 OR #5	20,586
7	#1 AND #2 AND #6	8,279

**Table 3:** Cochrane search

ID	Search	Result
#1	MeSH descriptor: (Breast Neoplasms) explode all trees	20,048
#2	(Breast cancer):ti,ab,kw	45,014
#3	MeSH descriptor: (Mastectomy) explode all trees	2,513
#4	(mastectomy):ti,ab,kw	6,398
#5	MeSH descriptor: (Mastectomy, Segmental) explode all trees	787
#6	(conservative breast surgery OR lumpectomy):ti,ab,kw	1,254
#7	#1 OR #2	46,435
#8	#3 OR #4	6,398
#9	#5 OR #6	1,779
#10	#7 AND #8 AND #9	1,259

**Table 4:** Embase search

No.	Query Results	Results
#1	‘breast cancer’/exp OR ‘breast cancer	728,291
#2	‘mastectomy’/exp OR ‘mastectomy’	85,432
#3	‘breast conserving surgery’/ exp OR ‘conservative breast surgery’ OR ‘lumpectomy’	29,851
#4	‘survival; OR ‘mortality’ OR ‘complications’	5,065,938
#5	#1 AND #2 AND #3 AND #4	10,029

Selection of Studies

Following an initial screening based on the title and abstract, two reviewers (CIMD and PS) independently selected trials for inclusion in this review using predetermined inclusion and exclusion criteria. This search used Rayyan [10] to extract relevant data, and duplicates were filtered. Keywords were employed to highlight inclusion and exclusion criteria-related words on Rayyan (Rayyan Systems Inc., Cambridge, MA). Any disagreement about the inclusion of the studies was resolved through consensus and consultation with a third reviewer (SSD). Subsequently, a full-text analysis was conducted with two reviewers (KLT and HO) who independently selected trials for inclusion in the review using predetermined inclusion and exclusion criteria. Disagreement about the inclusion of studies was resolved by consensus and consultation with a third review author (EC).

Data Extraction

The data extraction was performed by two independent reviewers, and disagreements were solved by consensus. When multiple overlapping reports from the same study were identified, the information from the one containing the most relevant information, or the first published report, was included.

Assessment of Risk of Bias in Included Studies

We evaluated the data using the criteria outlined in the Cochrane Handbook [11]. To assess the quality of observational studies included in the systematic review, we applied the Newcastle-Ottawa scale (NOS) to determine the risk of bias (Table 5) [12]. The NOS evaluates studies across three domains: the selection of study groups, comprising four items; the comparability of cohorts, consisting of one item; and the outcome of interest, encompassing three items. Within the selection and outcome domain, one star was allocated per item. The comparability domain permits a maximum allocation of two stars. Consequently, the highest achievable score on the NOS is 9 points. The final scores were interpreted as follows: good quality studies were identified as those achieving three or four stars in the selection domain, with one or two stars in the comparability domain and two or three stars in the outcome domain. Fair quality studies were granted two stars in the selection domain, one or two stars in comparability, and two or three stars in outcome. Poor quality studies were identified as those with 0 or one star in the selection domain, lacking stars in comparability, or receiving 0 or one star in the outcome domain. Two independent reviewers (TC and OYGG) evaluated the risk of bias in each study, considering the specific criteria and guidelines provided by the respective tools. Any reviewer discrepancies have been resolved through discussion with a third, blinded reviewer (EC).

**Table 5:** Risk of bias with the Newcastle-Ottawa scale (NOS) The NOS evaluates studies across three domains: the selection of study groups, comprising four items; the comparability of cohorts, consisting of one item; and the outcome of interest, encompassing three items. Within the selection and outcome domain, one star was allocated per item. The comparability domain permits a maximum allocation of two stars. Consequently, the highest achievable score on the NOS is 9 points. Good quality studies: ★★★ or ★★★★ in the selection domain, ★ or ★★ in the comparability domain, and ★★ or ★★★ in the outcome domain Fair quality studies: ★★ in the selection domain, ★ or ★★ in comparability, and ★★ or ★★★ in the outcome domain Poor quality studies: 0 or ★ in the selection domain, no star in comparability, or 0 or ★ in the outcome domain

No.	Author, Year	Study design	Section	Comparability	Outcome/Exposure	Total	Subjective evaluation
1	Li et al., 2019 [13]	Retrospective Cohort	★★★	★	★★★	7	Good Quality
2	Corradini et al., 2019 [14]	Retrospective Cohort	★★★★	★★	★★★	9	Good Quality
3	Xie et al., 2022 [15]	Retrospective Cohort	★★★	★★	★★★	8	Good Quality
4	Noh et al., 2008 [16]	Retrospective Cohort	★★★	★	★★★	7	Good Quality
5	Agarwal et al., 2014 [17]	Retrospective Cohort	★★★	★★	★★★	8	Good Quality
6	Christiansen et al., 2018 [18]	Retrospective Cohort	★★★	★★	★★★	8	Good Quality
7	Chu et al., 2021 [19]	Retrospective Cohort	★★★	★★	★★★	8	Good Quality
8	Yun et al., 2021 [20]	Retrospective Cohort	★★★★	★	★★★	8	Good Quality
9	Sang et al., 2022 [21]	Retrospective Cohort	★★★	★★	★★★	8	Good Quality
10	Li et al., 2022 [7]	Retrospective Cohort	★★★	★	★★★	7	Good Quality
11	Hartmann-Johnsen et al., 2015 [22]	Retrospective Cohort	★★★	★★	★★★	8	Good Quality
12	Hofvind et al., 2015 [23]	Retrospective Cohort	★★★	★★	★★★	8	Good Quality
13	de Boniface et al., 2021 [5]	Prospective Cohort	★★★	★★	★★★	8	Good Quality
14	Kim et al., 2016 [24]	Retrospective Cohort	★★★	★	★★★	7	Good Quality
15	Saifi et al., 2022 [25]	Retrospective Cohort	★★★	★★	★★★	8	Good Quality
16	Mburu et al., 2022 [26]	Retrospective Cohort	★★★	★★	★★★	8	Good Quality
17	Ghavami et al., 2023 [27]	Retrospective Cohort	★★★	★	★★★	7	Good Quality
18	Van Nguyen et al., 2021 [28]	Retrospective Cohort	★★★	★	★★★	7	Good Quality
19	Ye et al., 2015 [29]	Retrospective Cohort	★★★	★★	★★★	8	Good Quality
20	Onitilo et al., 2014 [30]	Retrospective Cohort	★★★	★★	★★★	8	Good Quality
21	Sayed et al., 2020 [31]	Retrospective Cohort	★★★	★★	★★★	8	Good Quality
22	Wang et al., 2018 [32]	Retrospective Cohort	★★★★	★★	★★	8	Good Quality

Statistical Analysis

A meta-analysis was performed using R (version 3.4.3; R Development Core Team, Vienna, Austria) [33]. The pooled effect of the outcomes was examined using a random-effects meta-analysis [34]. Whenever the number of studies reporting an outcome of interest was insufficient, only a qualitative analysis of the results was performed. Effect sizes were expressed as relative risk (RR) or standardized mean difference (SMD) and 95% confidence interval. The I² statistic assessed heterogeneity, and the following cut-off values were used for interpretation: <25, 25-50, and > 50% were considered small, medium, and large heterogeneity, respectively [35]. For all outcomes, sensitivity analyses according to the leave-one-out method were performed to determine the influence of individual studies on the overall effect, using diagnostic plots proposed by Viechtbauer et al. [36]. Egger's regression test examined publication bias when 10 or more reports with the same outcome were available [37]. Subgroup analysis will be performed by year, country, risk of bias, stage of breast cancer, and type of histological classification, if possible. To calculate the hazard ratio (HR) for overall survival (OS), we will extract the total sample size and the percentage of survivors from each study. Using these data, we estimated the number of events (non-survivors) and censored observations. We then applied the Parmar and Tierney methods to compute HRs from reconstructed Kaplan-Meier curves and summary survival statistics.

Results

Study Selection

**Figure 1:**
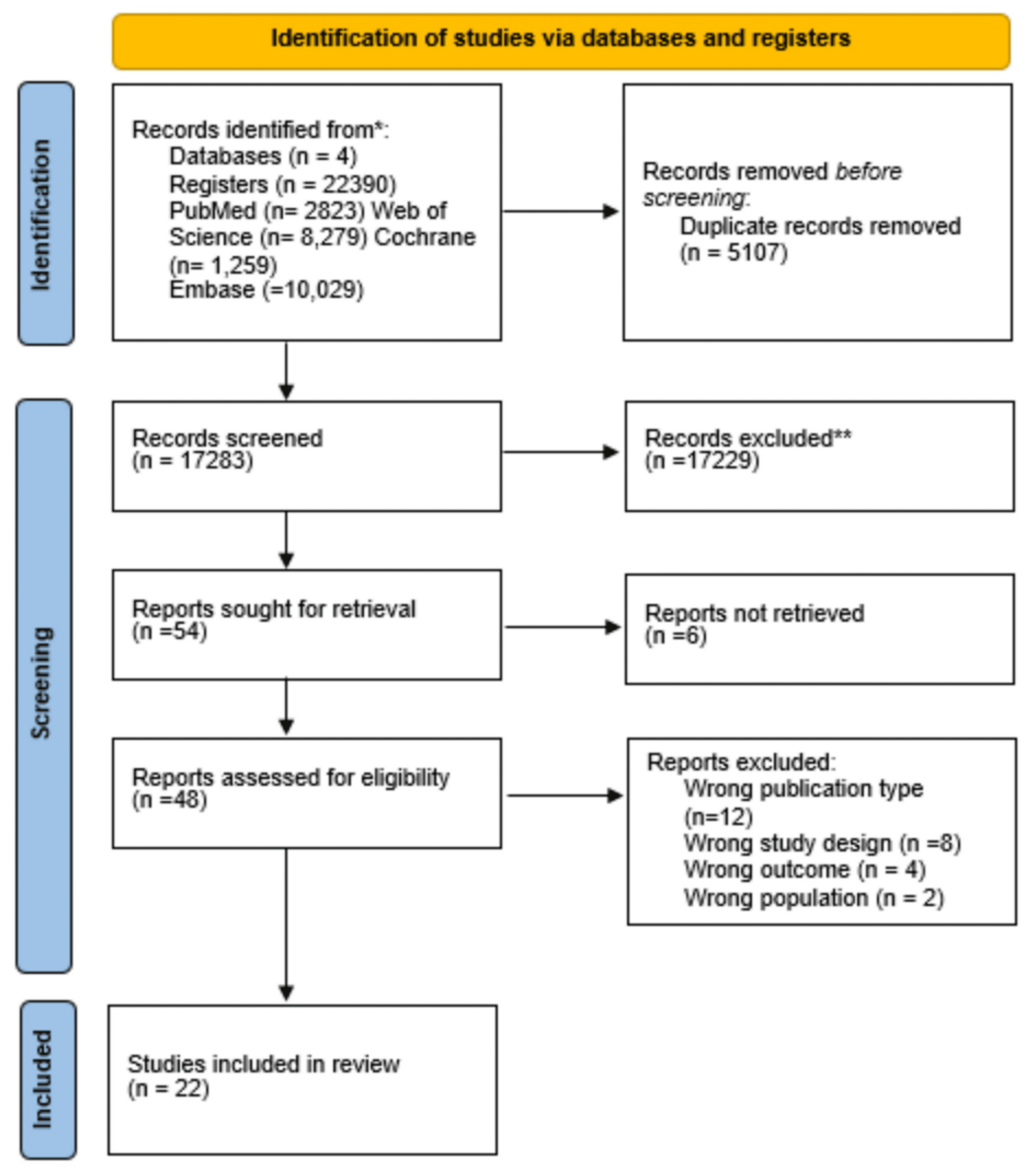
PRISMA flow diagram The Preferred Reporting Items for Systematic Reviews and Meta-Analyses (PRISMA) flow diagram delineates the systematic process of identifying and screening studies across multiple databases, culminating in selecting 22 pertinent studies.

Our initial search across four databases for observational studies yielded 22,390 potential articles. After eliminating 5,107 duplicates, we conducted screening based on title and abstract, leading to the exclusion of 17,229 articles. Six of the 54 articles sought for retrieval were not retrieved, leaving 48 articles for eligibility assessment. Twelve were excluded due to incorrect publication type, eight due to incorrect study design, four due to wrong outcomes, and two due to the wrong population. Ultimately, 22 studies were included in this review. This is summarized in our PRISMA flow chart (Figure 1).

Characteristics of the Included Studies

The 22 articles had a total sample size of 389,465 participants. The primary focus of the selected research papers is to compare BCS with MX in women with breast cancer, with emphasis on evaluating the safety and efficacy of each therapy through measures such as OS and breast cancer-specific survival (BCSS). The studies varied across a broad geographic range, which included these countries: China (28%), the USA (28%), Korea (14%), Norway (10%), Switzerland (4%), Sweden (4%), Denmark (4)%, Iran (4%), and Egypt (4%). While the selected studies presented varied results, most indicated improved survival outcomes with BCS with or without RT compared to MX. Approximately 71% of the studies reported enhanced survival using BCS, while the remaining 29% reported no survival benefit or significant difference. Despite reporting superior OS, BCSS, and disease-free survival (DFS) compared to MX alone, the reporting of these values was not consistent among studies. Therefore, while the trend towards better outcomes with BCS remained robust across various analyses, caution is warranted when comparing specific numerical results across studies due to these methodological differences. This information is summarized in Table 6.

**Table 6:** General outcomes BCS: Breast-conserving surgery; MX: Mastectomy; QOL: Quality of life; RT: Radiotherapy; TNBC: Triple Negative Breast Cancer; CT: Computed Tomography; BCSS: Breast cancer severity score; OS: overall survival; DFS: Disease-free survival; DDFS: Distant disease-free survival; United States of America

Author	Year	Country	Sample size	Study design	Intervention	Key Points
Li et al. [13]	2019	China	14,910	Retrospective cohort study	Breast-conserving surgery	BCS and MX are two mainstay treatments for early-stage breast cancer. BCS has been shown to be equivalent to MX. The study demonstrated that BCS for patients with T1-2N0M0 TNBC results in better survival than mastectomy, regardless of patient age, tumor grade, or tumor size. Patients with T1-2N0M0 TNBC should be considered for BCS. However, additional follow-up data and prospective studies are needed
Corradini et al. [14]	2019	Switzerland	7565	Retrospective cohort study	Breast-conserving surgery with RT	The present study showed that patients treated with BCS followed by RT had an improved outcome compared to MX alone. Specifically, local control, distant control, and overall survival were significantly better using the conservative approach. The present study shows that physicians should encourage patients to receive BCS with RT rather than MX, whenever it is medically feasible and appropriate
Xie et al. [15]	2022	China	44,755	Retrospective cohort study	Breast-conserving surgery	The study demonstrated that for elderly breast cancer patients, there is no difference in OS between BCS and MX despite clinicopathologic status, axillary surgical procedures or adjuvant therapies. In view of the condition of the elderly, it seems advisable to encourage all suitable patients over 70 years old to receive BCS rather than MX
Noh et al. [16]	2008	Korea	1174	Retrospective cohort study	Breast-conserving surgery with RT	The study showed a similar survival rate between BCS and MX while a tendency of more local recurrence in the BCS group for early breast cancer. We suggest that BCS can be a feasible alternative to MX in patients with pathologic T1-2N0 breast cancer
Agarwal et al. [17]	2014	USA	132,149	Retrospective cohort study	Breast-conserving surgery	Patients who underwent BCS have a higher BCSS rate compared with those treated with MX alone or MX with RT for early-stage invasive ductal carcinoma. Further investigation is warranted to understand what may be contributing to this effect
Christiansen et al. [18]	2017	Denmark	58,331	Retrospective cohort study	Breast-conserving surgery	Patients assigned to BCS have a better survival rate than patients assigned to MX. Residual confounding after adjustment for registered characteristics presumably explained the different outcomes, thus consistent with selection bias. Diversities in RT did not appear to explain the observed difference in survival after BCS and MX
Chu et al. [19]	2021	USA	18,260	Retrospective cohort study	Breast-conserving surgery with RT	Surgical approach, a factor that is within the control of the surgeon, has an impact on mortality for Louisiana women with early-stage breast cancer. BCS yielded better outcomes than MX, even after adjusting sociodemographic and clinical variables
Yun et al. [20]	2021	China	60	Retrospective cohort study	Breast-conserving surgery	Both BCS and modified radical mastectomy can remove tumor lesions in patients with early breast cancer. BCS is superior in reducing intraoperative blood loss, facilitating postoperative rehabilitation, relieving postoperative pain, protecting immune function and reducing postoperative complications, thereby improving the QOL of patients
Sang et al. [21]	2022	China	2080	Retrospective cohort study	Breast-conserving surgery	They showed comparable survival outcomes between patients receiving MX and BCS, and further supported the application of oncoplastic techniques in the surgical intervention following neoadjuvant chemotherapy
Li et al. [7]	2022	China	971	Retrospective cohort study	Breast-conserving surgery	This study suggests that there is no significant difference in BCSS between breast cancer patients undergoing BCS and MX after adjusting for confounding factors
Hartmann-Johnsen et al. [22]	2015	Norway	13,015	Retrospective cohort study	Breast-conserving surgery with RT	Survival was better or equal after BCS than MX in all early stages, surgical sub-cohorts, and age groups
Hofvind et al. [23]	2015	Norway	9547	Retrospective cohort study	Breast-conserving surgery	Showed a lower percentage of large tumors, lymph node positive tumors, histologic grade III tumors, Her2þ and Triple negative tumors among those who underwent CT compared with those who MX. And significantly higher six-year survival in women treated with BCS compared to MX, independent of prognostic and predictive tumor characteristics, age, detection mode and standard therapy
de Boniface et al. [5]	2021	Sweden	48,986	Prospective cohort study	Breast-conserving surgery with RT	BCS + RT is superior to MX with an overall BCSS gain of 56% to 70% in node negative patients
Kim et al. [24]	2016	Korea	286	Retrospective cohort study	Breast-conserving surgery with RT	MX patients are associated with a significantly higher risk of Loco-regional recurrence compared to those with BCS + RT among patients with T1-2/N1 breast cancer. TM was also associated with a significantly lower 5-year DFS rate compared with BCS+RT
Saifi et al. [25]	2022	USA	12,761	Retrospective cohort study	Breast-conserving surgery with RT	No representative clinical trials assessed the difference between BCS and MX in these patients. Our propensity-matched analysis study revealed that RT when added to lumpectomy is associated with better OS and BCSS compared to MX
Mburu et al. [26]	2022	USA	4333	Retrospective cohort study	Breast-conserving surgery with RT	Women who had BCS, MX or MX + RT had significantly higher hazard of death due to breast cancer compared with women who had BCS + RT. In this population-based study, women with TNBC treated with BCS + RT have a better prognosis compared with those treated with BCS, MX or MX + RT
Ghavami et al. [27]	2023	Iran	283	Retrospective cohort study	Breast-conserving surgery	Female patients with breast cancer showed that BCS was not related to improved DFS compared with MX
Van Nguyen et al. [28]	2021	Korea	428	Retrospective cohort study	Breast-conserving surgery	Patients in the BCS group exhibited approximately a 2.5-fold increased risk of LR compared with those in the MX alone group one-third of patients with an isolated LR after BCS experienced distant metastasis despite of aggressive salvage MX followed by systemic treatments. Although BCS had equivalent OS to total MX alone and it can be recognized as the first local treatment option for young women with breast cancer, countermeasures are required
Ye et al. [29]	2015	USA	7665	Retrospective cohort study	Breast-conserving surgery with RT	The BCS group continued to show non-inferior BCSS and OS compared to the MX group in all of the subgroups. This study suggests that while young age may be a poor prognostic factor for breast cancer, there is no evidence that these patients have better outcomes with MX over BCS and continues to support the use of BCS there are many other factors (cosmesis, ha, long term toxicities etc.) that must be considered in deciding between BCS and MX. If there are reasons patients should not or cannot undergo RT, patients should undergo MX, as RT is an integral part of BCS
Onitilo et al. [30]	2014	USA	5335	Retrospective cohort study	Breast-conserving surgery with RT	The study showed no difference in the OS by breast cancer surgery type when the effects of adjuvant RT and other covariates were eliminated using statistical methods. Comparison of BCS plus RT to MX alone revealed a significant survival benefit with BCS, suggesting that the prognostic differences reported here. Breast cancer surgery and survival
Sayed et al. [31]	2020	Egypt	434	Retrospective cohort study	Breast-conserving surgery with RT	CBT resulted in better DDFS, DFS, and OS than MRM. Conflicting results have been reported regarding RT being the reason for the significantly better survival associated with CBT versus MX
Wang et al. [32]	2018	China	6137	Retrospective cohort study	Breast-conserving surgery with RT	BCS plus RT does not compromise local control compared with MX in Stage I breast cancer. BCS plus RT provides better long-term OS and distant metastasis-free survival compared with MX in Stage I breast cancer. BCS plus RT should be preferred for patients with Stage I breast cancer

Risk of Bias Within Studies

The risk of bias assessment utilized the NOS for all 22 cohort studies [12]. Our results in Table 5 show that all 22 studies were deemed good quality. A good quality study strongly correlates with a low risk of bias, as the rigorous methodological criteria evaluated by the scale minimize systematic errors and enhance the reliability of study findings, thereby indicating a reduced likelihood of bias.

Regional Recurrence

This meta-analysis assessed the effect of various interventions on regional recurrence in breast cancer. Four studies with a total of 4,511 participants (1,327 in the experimental group and 3,184 in the control group) were included. The random-effects model indicated an odds ratio (OR) of 1.13 (95% CI: 0.26-5.03, p = 0.87, I² = 83.8%). The prediction interval ranged from 0.0012 to 1088.41, highlighting the wide potential variation in new studies.

Publication Bias

The funnel plot for regional recurrence outcomes was asymmetric, indicating the possibility of publication bias. Due to the limited number of studies, Egger's test for small-study effects was not possible and could not be conclusively interpreted.

Subgroup and Sensitivity Analysis

Subgroup analysis by country revealed significant differences (p = 0.047), with studies from Korea reporting an OR of 0.39 (95% CI: 0.22-0.70, I² = 0.0%) and studies from China showing an OR of 4.07 (95% CI: 0.43-38.16, I² = 77.0%). Analysis by intervention also revealed significant differences (p = 0.047), with BCS with RT (OR = 0.39, 95% CI: 0.22-0.70, I² = 0.0%) vs. BCS alone (OR = 4.07, 95% CI: 0.43-38.16, I² = 77.0%). Analysis by type of breast cancer showed significant differences for unspecified types (OR = 0.44, 95% CI: 0.21-0.95, p = 0.0003). Analysis by stage of breast cancer also showed significant differences for stages I, IIA (OR = 0.44, 95% CI: 0.21-0.95, p = 0.0003). Other subgroup analyses by year and study design did not show significant differences. The sensitivity analysis identified Noh et al. [16], Sang et al. [21], and Kim et al. [24] as potential outliers. Omitting these studies significantly impacted heterogeneity and the overall effect. However, excluding these outliers was not feasible as it would leave only one study in the meta-analysis (Tables 7-8).

**Table 7:** Regional recurrence - analysis of subgroups --: Not available/applicable

Subgroup	k	Effect Size (OR) (95%CI)	I^2^ (%)	p-value (subgroup difference)
RoB (Risk of Bias)				
Low	4	1.1339 (0.2556; 5.0305)	83.8	--
Country				
Korea	2	0.3893 (0.2154; 0.7038)	0	0.047
China	2	4.0690 (0.4339; 38.1550)	77	--
Year				
2008	1	0.4448 (0.2076; 0.9529)	--	0.1199
2022	2	4.0690 (0.4339; 38.1550)	77	--
2016	1	0.3179 (0.1241; 0.8141)	--	--
Study Design				
Cohort study	4	1.1339 (0.2556; 5.0305)	83.8	--
Intervention				
Breast-conserving surgery with RT	2	0.3893 (0.2154; 0.7038)	0	0.047
Breast-conserving surgery	2	4.0690 (0.4339; 38.1550)	77	--
Type of Breast Cancer				
Not specified	1	0.4448 (0.2076; 0.9529)	--	0.0003
Early breast cancer	1	1.5916 (0.8596; 2.9467)	--	--
Multiple	1	16.3253 (1.9995; 133.2904)	--	--
Invasive breast cancer	1	0.3179 (0.1241; 0.8141)	--	--
Stage of Breast Cancer				
I, IIA	1	0.4448 (0.2076; 0.9529)	--	0.0003
II, III	1	1.5916 (0.8596; 2.9467)	--	--
I, II, III, IV	1	16.3253 (1.9995; 133.2904)	--	--
I, II	1	0.3179 (0.1241; 0.8141)	--	--

**Table 8:** Regional recurrence - leave-one-out analysis The following are different influence diagnostics used in meta-analysis (specifically in R's meta for package) to detect studies that might unduly influence the overall results: RR: regional recurrence; *: studies flagged as influential; rstudent: studentized residual – identifies outliers based on residuals; dffits: influence of a study on its predicted value (high values = high influence); cook.d: Cook’s distance – influence on overall model fit; cov.r: covariance ratio – influence on precision of estimates; tau^2^.del: between-study variance (τ²) with study deleted; QE.del: heterogeneity statistic (Q) with study deleted; hat: hat value (leverage) – influence of a study on its fitted value; Hat: duplicate of hat, possibly different formatting in table; Weight: study weight in the meta-analysis (typically inverse-variance based); is.infl: TRUE/FALSE flag indicating influential studies; Is Influential: same as is.infl.

Author	RR	Lower 95%CI	Upper 95%CI	I^2^	rstudent	dffits	cook.d	cov.r	tau^2^.del	QE.del	hat	Weight	Influential	is.infl
Omitting Noh et al. [16]	1.2142	0.7568	1.9478	0.8610	-2.0447	-1.3429	1.8033	1.4313	0.0000	14.3837	0.3014	30.1353	*	Yes
Omitting Sang et al. [21]	0.6427	0.3857	1.0708	0.8270	2.6464	2.4480	5.9927	1.8557	0.0000	11.5609	0.4611	46.1109	*	Yes
Omitting Li et al. [7]	0.7172	0.4648	1.1067	0.8136	2.7989	0.5689	0.3237	1.0413	0.0000	10.7306	0.0397	3.9677	No	No
Omitting Kim et al. [24]	1.1565	0.7427	1.8008	0.8479	-2.3278	-1.1561	1.3366	1.2467	0.0000	13.1460	0.1979	19.7861	*	Yes

Local Recurrence

This meta-analysis assessed the effect of various interventions on local recurrence in breast cancer. Six studies with a total of 12,504 participants (8,050 in the experimental group and 4,454 in the control group) were included. The random-effects model indicated an OR of 1.65 (95% CI: 0.79-3.47, p = 0.19, I² = 70.6%). The prediction interval ranged from 0.15 to 17.68, highlighting the wide potential variation in new studies.

Publication Bias

The funnel plot for local recurrence outcomes was asymmetric, indicating the possibility of publication bias. Due to the limited number of studies, Egger's test for small-study effects was not possible and could not be conclusively interpreted.

Subgroup and Sensitivity Analysis

Subgroup analysis by year revealed significant differences (p = 0.0101), with studies from 2019 reporting an OR of 1.68 (95% CI: 1.27-2.22). Analysis by type of breast cancer also showed significant differences for invasive breast cancer (OR = 1.68, 95% CI: 1.27-2.22, p = 0.0110). Other subgroup analyses by country, study design, intervention, and stage of breast cancer did not show significant differences. The sensitivity analysis identified Corradini et al. [14] and Kim et al. [24] as potential outliers. Omitting these studies significantly impacted heterogeneity and the overall effect. Excluding these outliers resulted in an OR of 2.21 (95% CI: 1.34-3.65, p = 0.0019, I² = 23.7%), indicating a more consistent effect across the remaining studies (Tables 9-10).

**Table 9:** Local recurrence - analysis of subgroups --: Not available/applicable

Subgroup	k	Effect Size (OR) (95%CI)	I^2^ (%)	p-value (subgroup difference)
RoB (Risk of Bias)				
Low	6	1.6515 (0.7854; 3.4726)	70.6	--
Country				
Switzerland	1	1.6836 (1.2740; 2.2249)	--	0.5587
Korea	3	1.1503 (0.3280; 4.0335)	82	--
China	2	5.9726 (0.3800; 93.8681)	71.2	--
Year				
2019	1	1.6836 (1.2740; 2.2249)	--	0.0101
2008	1	1.8603 (0.9168; 3.7746)	--	--
2022	2	5.9726 (0.3800; 93.8681)	71.2	--
2016	1	0.3179 (0.1241; 0.8141)	--	--
2021	1	2.5772 (0.8796; 7.5510)	--	--
Study Design				
Cohort Study	6	1.6515 (0.7854; 3.4726)	70.6	--
Intervention				
Breast-conserving surgery with RT	3	1.0639 (0.3720; 3.0420)	82.6	0.1616
Breast-conserving surgery	3	2.6303 (1.2942; 5.3457)	42.4	--
Type of Breast Cancer				
Invasive breast cancer	1	1.6836 (1.2740; 2.2249)	--	0.011
Not specified	1	1.8603 (0.9168; 3.7746)	--	--
Early breast cancer	1	1.9542 (0.7193; 5.3090)	--	--
Multiple	2	6.6781 (0.5697; 78.2817)	64.2	--
Invasive breast cancer	1	0.3179 (0.1241; 0.8141)	--	--
Stage of Breast Cancer				
I, II	2	0.7791 (0.1528; 3.9725)	91	0.2512
I, IIA	1	1.8603 (0.9168; 3.7746)	--	--
II, III	1	1.9542 (0.7193; 5.3090)	--	--
I, II, III, IV	1	35.0000 (1.9924; 614.8434)	--	--
I, II	1	2.5772 (0.8796; 7.5510)	--	--

**Table 10:** Local recurrence - leave-one-out analysis The following are different influence diagnostics used in meta-analysis (specifically in R's meta for package) to detect studies that might unduly influence the overall results: rstudent: studentized residual – identifies outliers based on residuals; dffits: influence of a study on its predicted value (high values = high influence); cook.d: Cook’s distance – influence on overall model fit; cov.r: covariance ratio – influence on precision of estimates; tau2.del: between-study variance (τ²) with study deleted; QE.del: heterogeneity statistic (Q) with study deleted; hat: hat value (leverage) – influence of a study on its fitted value; Hat: duplicate of hat, possibly different formatting in table; Weight: study weight in the meta-analysis (typically inverse-variance based); is.infl: TRUE/FALSE flag indicating influential studies; Is Influential: same as is.infl.

Author	RR	Lower 95%CI	Upper 95%CI	I^2^	rstudent	dffits	cook.d	cov.r	tau2.del	QE.del	Hat	Weight	Is Influential	is.infl
Omitting Corradini et al. [14]	1.585530	1.055323	2.382119	0.759963	0.5856242	0.9287212	0.8625230	3.514970	0	16.664070	0.7155025	71.550251	Yes	Yes
Omitting Noh et al. [16]	1.633819	1.281364	2.083222	0.762294	0.4236555	0.1497476	0.0224244	1.124938	0	16.827542	0.1110621	11.106207	No	No
Omitting Sang et al. [21]	1.643987	1.299013	2.080576	0.762676	0.3903958	0.0947844	0.0089841	1.058947	0	16.854617	0.0556659	5.566588	No	No
Omitting Li et al. [7]	1.577035	1.250445	1.988925	0.681116	2.1126473	0.1744080	0.0304181	1.006815	0	12.543747	0.0067691	0.676907	No	No
Omitting Kim et al. [24]	1.843792	1.444935	2.352751	0.166910	-3.4936547	-0.9050180	0.8190575	1.067105	0	4.801402	0.0628850	6.288504	Yes	Yes
Omitting Van Nguyen et al. [28]	1.614668	1.275584	2.043888	0.753619	0.8786274	0.1975399	0.0390220	1.050548	0	16.235039	0.0481154	4.811543	No	No

DFS at Five Years

This meta-analysis evaluated the effect of various interventions on DFS at five years in breast cancer. Eight studies with a total of 12,504 participants (8,050 in the experimental group and 4,454 in the control group) were included. The random-effects model indicated an HR of 0.78 (95% CI: 0.57 to 1.09, p = 0.14, I² = 98.2%). The prediction interval ranged from 0.25 to 2.44, highlighting the wide potential variation in new studies.

Publication Bias

The funnel plot for DFS5y outcomes was asymmetric, indicating the possibility of publication bias. Due to the limited number of studies, Egger's test for small-study effects was not possible and could not be conclusively interpreted.

Subgroup and Sensitivity Analysis

Subgroup analysis by year revealed significant differences (p < 0.0001), particularly for studies from 2019 (HR = 0.85, 95% CI: 0.74-0.97) and triple-negative breast cancer (HR = 0.85, 95% CI: 0.74-0.97, p < 0.0001). Subgroup analysis by type of breast cancer revealed significant differences (p < 0.0001) for triple-negative breast cancer (HR = 0.85, 95% CI: 0.74-0.97). Other subgroup analyses for invasive breast cancer, early breast cancer, and multiple types did not show significant differences. Subgroup analysis by stage of breast cancer also revealed significant differences (p < 0.0001) for stages I, IIA (HR = 0.85, 95% CI: 0.74-0.97). Other stages, such as I and II and I, II, and III, did not show significant differences. Other subgroup analyses by country, study design, and intervention did not show significant differences. The sensitivity analysis identified Li et al. [13], Agarwal et al. [17], Mburu et al. [26], and Ye et al. [29] as potential outliers. Omitting these studies significantly impacted heterogeneity and the overall effect. Excluding these outliers resulted in an HR of 0.60 (95% CI: 0.48-0.76, p < 0.0001, I² = 46.5%), indicating a more consistent effect across the remaining studies (Tables 11-12).

**Table 11:** Disease-free survival at five years - analysis of subgroups --: Not available/applicable

Subgroup	k	Effect Size (HR) (95%CI)	I^2^ (%)	p-value (subgroup difference)
RoB (Risk of Bias)				
Low	8	0.7842 (0.5657; 1.0871)	98.2	--
Country				
China	2	0.6470 (0.3692; 1.1340)	92.6	0.711
USA	4	0.8468 (0.5020; 1.4285)	99.2	--
Korea	1	0.6705 (0.3441; 1.3062)	--	--
Egypt	1	1.3500 (0.3716; 4.9051)	--	--
Year				
2019	1	0.8507 (0.7425; 0.97470	--	< 0.0001
2014	1	0.5000 (0.4718; 0.5299)	--	--
2021	1	0.6518 (0.5887; 0.7216)	--	--
2016	1	0.6705 (0.3441; 1.3062)	--	--
2022	1	0.9264 (0.8044; 1.0668)	--	--
2015	1	1.7143 (1.5233; 1.9292)	--	--
2020	1	1.3500 (0.3716; 4.9051)	--	--
2018	1	0.4796 (0.3646; 0.6309)	--	--
Study Design				
Cohort Study	8	0.7842 (0.5657; 1.0871)	98.2	--
Intervention				
Breast-conserving surgery	2	0.6498 (0.3860; 1.0939)	98	0.435
Breast-conserving surgery with RT	6	0.8477 (0.5584; 1.2868)	97.1	--
Type of Breast Cancer				
Triple negative breast cancer	1	0.8507 (0.7425; 0.9747)	--	< 0.0001
Invasive breast cancer	2	0.5011 (0.4729; 0.5309)	0	--
Early breast cancer	1	0.6518 (0.5887; 0.7216)	--	--
Triple-Negative Breast Cancer (	1	0.9264 (0.8044; 1.0668)	--	--
Multiple	3	0.9957 (0.4123; 2.4042)	97.1	--
Stage of Breast Cancer				
I, IIA	1	0.8507 (0.7425; 0.9747)	--	< 0.0001
I, II	3	0.5771 (0.4612; 0.7222)	90.1	--
I, II, III	2	0.6739 (0.3536; 1.2841)	94.3	--
I, IIA, IIB	1	1.7143 (1.5233; 1.9292)	--	--
I, II	1	1.3500 (0.3716; 4.9051)	--	--

**Table 12:** Disease-free survival at five years - leave-one-out analysis The following are different influence diagnostics used in meta-analysis (specifically in R's meta for package) to detect studies that might unduly influence the overall results: rstudent: studentized residual – identifies outliers based on residuals; dffits: influence of a study on its predicted value (high values = high influence); cook.d: Cook’s distance – influence on overall model fit; cov.r: covariance ratio – influence on precision of estimates; tau2.del: between-study variance (τ²) with study deleted; QE.del: heterogeneity statistic (Q) with study deleted; hat: hat value (leverage) – influence of a study on its fitted value; Hat: duplicate of hat, possibly different formatting in table; Weight: study weight in the meta-analysis (typically inverse-variance based); is.infl: TRUE/FALSE flag indicating influential studies; Is Influential: same as is.infl.

Author	Effect	Lower	Upper	I^2^	rstudent	dffits	cook.d	cov.r	tau2.del	QE.del	Hat	Weight	Is Influential
Omitting Li et al. [13]	-0.41911052	-0.4624942	-0.3757268	0.983669	3.5343912	1.127244e+00	1.270680e+00	1.101720	0	367.3880	0.0923285	9.2328485	Yes
Omitting Agarwal et al. [17]	-0.08925907	-0.1481164	-0.0304017	0.965697	-14.3166998	-1.451415e+01	2.106607e+02	2.027774	0	174.9120	0.5068484	50.684838	Yes
Omitting Chu et al. [19]	-0.38887639	-0.4341089	-0.3436438	0.984186	-0.6893379	-3.064438e-01	9.390780e-02	1.197623	0	379.4047	0.1650126	16.501264	No
Omitting Kim et al. [24]	-0.39532159	-0.4367336	-0.3539096	0.984206	-0.0131339	-8.155254e-04	6.650816e-07	1.003856	0	379.8798	0.0038408	0.3840759	No
Omitting Mburu et al. [26]	-0.42522959	-0.4684560	-0.3820031	0.983261	4.6292654	1.417408e+00	2.009044e+00	1.093749	0	358.4498	0.0857132	8.5713216	Yes
Omitting Ye et al. [29]	-0.52577096	-0.5698941	-0.4816478	0.943315	16.5539225	6.185031e+00	3.825461e+01	1.139599	0	105.8476	0.1224983	12.249827	Yes
Omitt ing Sayed et al. [31]	-0.39605327	-0.4374069	-0.3546996	0.984159	1.0570277	3.388081e-02	1.147909e-03	1.001027	0	378.7626	0.0010263	0.1026334	No
Omitting Wang et al. [32]	-0.38744223	-0.4292526	-0.3456319	0.983951	-2.4551830	-3.744510e-01	1.402135e-01	1.023261	0	373.8520	0.0227319	2.2731909	No

DFS at 10 Years

This meta-analysis evaluated the effect of various interventions on DFS in breast cancer. Five studies with a total of participants were included. The random-effects model indicated a hazard ratio (HR) of 1.12 (95% CI: 0.79 to 1.58, p = 0.53, I² = 96.4%). The prediction interval ranged from 0.32 to 3.93, highlighting the wide potential variation in new studies.

Publication Bias

The funnel plot for DFS10 years outcomes was asymmetric, indicating the possibility of publication bias. Due to the limited number of studies, Egger's test for small-study effects was not possible and could not be conclusively interpreted.

Subgroup and Sensitivity Analysis

Subgroup analysis by country revealed significant differences (p < 0.0001) for studies from Korea (HR = 1.28, 95% CI: 0.97-1.69, I² = 0.0%). Subgroup analysis by stage of breast cancer also revealed significant differences (p < 0.0001) for stages I and IIA (HR = 1.27, 95% CI: 0.93-1.72). Other subgroup analyses by year, study design, intervention, type of breast cancer, and other stages of breast cancer did not show significant differences. The sensitivity analysis identified Hartmann-Johnsen et al. [22] and Ye et al. [29] as potential outliers. Omitting these studies significantly impacted heterogeneity and the overall effect. Excluding these outliers resulted in an HR of 1.10 (95% CI: 0.79-1.52, p = 0.59, I² = 42.3%), indicating a more consistent effect across the remaining studies (Tables 13-14).

**Table 13:** Disease-free survival at 10 years - analysis of subgroups --: Not available/applicable

Subgroup	k	Effect Size (HR) (95%CI)	I^2^ (%)	p-value (subgroup difference)
RoB (Risk of Bias)				
Low	5	1.1161 (0.7908; 1.5751)	96.4	--
Country				
Korea	2	1.2835 (0.9720; 1.6948)	0.0	< 0.0001
China	1	0.8125 (0.5466; 1.2077)	--	--
Norway	1	0.7500 (0.6777; 0.8301)	--	--
USA	1	1.7143 (1.5233; 1.9292)	--	--
Year				
2008	1	1.2667 (0.9320; 1.7215)	--	0.3247
2022	1	0.8125 (0.5466; 1.2077)	--	--
2015	2	1.1332 (0.5041; 2.5477)	99.1	--
2021	1	1.3636 (0.7066; 2.6317)	--	--
Study Design				
Cohort Study	5	1.1161 (0.7908; 1.5751)	96.4	--
Intervention				
Breast-conserving surgery with RT	3	1.1725 (0.7181; 1.9144)	98.2	0.6163
Breast-conserving surgery	2	0.9822 (0.6024; 1.6017)	42.8	--
Type of Breast Cancer				
Not specified	1	1.2667 (0.9320; 1.7215)	--	0.5578
Multiple	4	1.0812 (0.7022; 1.6648)	97.3	--
Stage of Breast Cancer				
I, IIA	1	1.2667 (0.9320; 1.7215)	--	< 0.0001
I, II, III, IV	1	0.8125 (0.5466; 1.2077)	--	--
I, II, III	1	0.7500 (0.6777; 0.8301)	--	--
I, II	1	1.3636 (0.7066; 2.6317)	--	--
I, IIA, IIb	1	1.7143 (1.5233; 1.9292)	--	--

**Table 14:** Disease-free survival at 10 years - leave-one-out analysis The following are different influence diagnostics used in meta-analysis (specifically in R's meta for package) to detect studies that might unduly influence the overall results: rstudent: studentized residual – identifies outliers based on residuals; dffits: influence of a study on its predicted value (high values = high influence); cook.d: Cook’s distance – influence on overall model fit; cov.r: covariance ratio – influence on precision of estimates; tau2.del: between-study variance (τ²) with study deleted; QE.del: heterogeneity statistic (Q) with study deleted; hat: hat value (leverage) – influence of a study on its fitted value; Hat: duplicate of hat, possibly different formatting in table; Weight: study weight in the meta-analysis (typically inverse-variance based); is.infl: TRUE/FALSE flag indicating influential studies; Is Influential: same as is.infl.

Author	Effect	Lower	Upper	I^2^	rstudent	dffits	cook.d	cov.r	tau2.del	QE.del	Hat	Weight	Is Influential
Omitting Noh et al. [26]	0.05671028	-0.018324	0.1317445	0.972887	1.1150695	0.27273191	0.07438270	1.059823	0	110.65025	0.05644620	5.644620	No
Omitting Li et al. [7]	0.07645981	0.0023095	0.1506102	0.972724	-1.3809303	-0.25835028	0.06674487	1.035001	0	109.98667	0.03381690	3.381690	No
Omitting Hartmann-Johnsen et al. [22]	0.44562542	0.3408024	0.5504484	0.797122	-9.8542581	-10.18554219	103.7453000	2.068367	0	14.78723	0.51652679	51.652679	Yes
Omitting Van Nguyen et al. [28]	0.06382507	-0.0095128	0.1371629	0.973061	0.7298137	0.08140898	0.00662742	1.012443	0	111.36101	0.01228995	1.228995	No
Omitting Ye et al. [29]	-0.22365804	-0.3162919	-0.1310242	0.763937	9.9591746	7.81208567	61.0286800	1.615301	0	12.70848	0.38092016	38.092016	Yes

Overall Survival at Five Years

This meta-analysis evaluated the effect of various interventions on overall survival at five years in breast cancer. Ten studies with a total of participants were included. The random-effects model indicated an HR of 0.49 (95% CI: 0.34-0.71, p = 0.0001, I² = 98.8%). The prediction interval ranged from 0.12 to 1.97, highlighting the wide potential variation in new studies.

Publication Bias

The funnel plot for OS5 outcomes was asymmetric, and Egger's linear regression test did not indicate significant asymmetry (t = 0.14, df = 8, p = 0.8941).

Subgroup and Sensitivity Analysis

Subgroup analysis by country revealed significant differences (p < 0.0001) for studies from China (HR = 0.42, 95% CI: 0.17-1.06, I² = 90.5%). Subgroup analysis by year revealed significant differences (p < 0.0001) for studies from 2019 (HR = 0.62, 95% CI: 0.55-0.71, I² = 30.0%). Subgroup analysis by type of breast cancer showed significant differences (p < 0.0001) for triple-negative breast cancer (HR = 0.65, 95% CI: 0.59-0.71). Subgroup analysis by stage of breast cancer also revealed significant differences (p < 0.0001) for stages I and IIA (HR = 0.65, 95% CI: 0.59-0.71). Other subgroup analyses by study design and intervention.

The sensitivity analysis identified Li et al. [13], Hartmann-Johnsen et al. [22], de Boniface et al. [5], Saifi et al. [25], Ye et al. [29], and Onitilo et al. [30] as potential outliers. Omitting these studies significantly impacted heterogeneity and the overall effect. Excluding these outliers resulted in an HR of 0.42 (95% CI: 0.30-0.59, p < 0.0001, I² = 60.7%), indicating a more consistent effect across the remaining studies (Tables 15-16). 

**Table 15:** Overall survival at five years - analysis of subgroups --: Not available/applicable

Subgroup	k	Effect Size (HR) (95%CI)	I^2^ (%)	p-value (subgroup difference)
RoB (Risk of Bias)				
Low	10	0.4877 (0.3366; 0.7068)	98.8	--
Country				
China	2	0.4197 (0.1659; 1.0618)	90.5	< 0.0001
Switzerland	1	0.5556 (0.4410; 0.6998)	--	--
Norway	1	0.2500 (0.2213; 0.2824)	--	--
Sweden	1	0.3333 (0.3102; 0.3582)	--	--
USA	4	0.6897 (0.3630; 1.3105)	99.0	--
Egypt	1	0.3333 (0.1141; 0.9741)	--	--
Year				
2019	2	0.6225 (0.5467; 0.7089)	30.0	< 0.0001
2015	2	0.6512 (0.0998; 4.2505)	99.8	--
2021	1	0.3333 (0.3102; 0.3582)	--	--
2022	2	0.5708 (0.3474; 0.937)	96.0	--
2014	1	0.4091 (0.3490; 0.4796)	--	--
2020	1	0.3333 (0.1141; 0.974)	--	--
2018	1	0.2500 (0.1420; 0.440)	--	--
Study Design				
Cohort Study	10	0.4877 (0.3366; 0.706)	98.8	--
Intervention				
Breast-conserving surgery	1	0.6471 (0.5878; 0.712)	--	0.1391
Breast-conserving surgery with RT	9	0.4704 (0.3117; 0.7099)	98.9	--
Type of Breast Cancer				
Triple-negative breast cancer	1	0.6471 (0.5878; 0.7123)	--	< 0.0001
Invasive breast cancer	1	0.5556 (0.4410; 0.6998)	--	--
Multiple	6	0.4672 (0.2464; 0.8858)	99.3	--
Triple-negative breast cancer	1	0.4419 (0.3776; 0.5170)	--	--
Not specified	1	0.4091 (0.3490; 0.4796)	--	--
Stage of Breast Cancer				
I, IIA	1	0.6471 (0.5878; 0.7123)	--	< 0.0001
I, II	2	0.4251 (0.2578; 0.7009)	94.2	--
I, II, III	4	0.3869 (0.2311; 0.6478)	98.1	--
I, IIA, IIb	1	1.6957 (1.5114; 1.9023)	--	--
I, II, III, IV	1	0.4091 (0.3490; 0.4796)	--	--
I, II	1	0.3333 (0.1141; 0.9741)	--	--

**Table 16:** Overall survival at five years - leave-one-out analysis The following are different influence diagnostics used in meta-analysis (specifically in R's meta for package) to detect studies that might unduly influence the overall results: rstudent: studentized residual – identifies outliers based on residuals; dffits: influence of a study on its predicted value (high values = high influence); cook.d: Cook’s distance – influence on overall model fit; cov.r: covariance ratio – influence on precision of estimates; tau2.del: between-study variance (τ²) with study deleted; QE.del: heterogeneity statistic (Q) with study deleted; hat: hat value (leverage) – influence of a study on its fitted value; Hat: duplicate of hat, possibly different formatting in table; Weight: study weight in the meta-analysis (typically inverse-variance based); is.infl: TRUE/FALSE flag indicating influential studies; Is Influential: same as is.infl.

Author	Effect	Lower	Upper	I^2^	rstudent	dffits	cook.d	cov.r	tau2.del	QE.del	Hat	Weight	Is Influential
Omitting Li et al. [13]	-0.7310814	-0.7754189	-0.6867438	0.989061	5.4800315	2.52987016	6.400243e+00	1.213123	0	731.3488	0.175681288	17.5681288	Yes
Omitting Corradini et al. [13]	-0.6819856	-0.7228669	-0.6411043	0.989484	0.7875062	0.13945819	1.944859e-02	1.031360	0	760.7594	0.030406732	3.0406732	No
Omitting Hartmann-Johnsen et al. [22]	-0.5926835	-0.6353278	-0.5500392	0.987023	-12.0376896	-4.20854620	1.771186e+01	1.122230	0	616.4735	0.108917090	10.8917090	Yes
Omitting de Boniface et al. [5]	-0.4880195	-0.5365857	-0.4394533	0.985998	-13.7854872	-9.30450180	8.657375e+01	1.455556	0	571.3399	0.312977426	31.2977426	Yes
Omitting Saifi et al. [25]	-0.7262441	-0.7689924	-0.6834957	0.988891	6.4200346	2.29434912	5.264038e+00	1.127716	0	720.1627	0.113251679	11.3251679	Yes
Omitting Mburu et al. [26]	-0.6694519	-0.7110968	-0.6278069	0.989449	-1.7762370	-0.47079210	2.216452e-01	1.070252	0	758.2245	0.065640306	6.5640306	No
Omitting Ye et al. [29]	-0.8476667	-0.8906401	-0.8046934	0.971331	21.9621349	8.20626394	6.734277e+01	1.139618	0	279.0441	0.122513091	12.2513091	Yes
Omitting Onitilo et al. [30]	-0.6644068	-0.7060183	-0.6227952	0.989388	-2.7366182	-0.71643174	5.132744e-01	1.068536	0	753.8904	0.064140433	6.4140433	Yes
Omitting Sayed et al. [31]	-0.6785294	-0.7188127	-0.6382460	0.989485	-0.7672278	-0.02882024	8.306061e-04	1.001411	0	760.7909	0.001409075	0.1409075	No
Omitting Wang et al. [32]	-0.6755227	-0.7158800	-0.6351655	0.989409	-2.4561513	-0.17520886	3.069814e-02	1.005089	0	755.3468	0.005062879	0.5062879	No

Overall Survival at 10 Years

This meta-analysis evaluated the effect of various interventions on overall survival at 10 years in breast cancer. Nine studies with a total of participants were included. The random-effects model indicated an HR of 0.62 (95% CI: 0.42-0.91, p = 0.0149, I² = 98.8%). The prediction interval ranged from 0.15 to 2.60, highlighting the wide potential variation in new studies.

Publication Bias

The funnel plot for OS10 outcomes was asymmetric, and Egger's test was not possible due to the limited number of studies.

Subgroup and Sensitivity Analysis

Subgroup analysis by country revealed significant differences (p < 0.0001) for studies from Switzerland (HR = 0.59, 95% CI: 0.51-0.69). Subgroup analysis by year also showed significant differences (p < 0.0001) for studies from 2019 (HR = 0.59, 95% CI: 0.51-0.69). Subgroup analysis by stage of breast cancer revealed significant differences (p < 0.0001) for stages I and IIA (HR = 0.65, 95% CI: 0.59-0.71). Other subgroup analyses by study design, intervention, and type of breast cancer did not show significant differences.

The sensitivity analysis identified Christiansen et al. [18], Chu et al. [19], Hartmann-Johnsen et al. [22], de Boniface et al. [5], and Ye et al. [29] as potential outliers. Omitting these studies significantly impacted heterogeneity and the overall effect. Excluding these outliers resulted in an HR of 0.66 (95% CI: 0.35-1.25, p = 0.1997, I² = 80.2%), indicating a more consistent effect across the remaining studies (Tables 17-18).

**Table 17:** Overall survival at 10 years - analysis of subgroups --: Not available/applicable

Subgroup	k	Effect Size (HR) (95%CI)	I^2^ (%)	p-value (subgroup difference)
RoB (Risk of Bias)				
Low	9	0.6151 (0.4159; 0.9097)	98.8	--
Country				
Switzerland	1	0.5909 (0.5050; 0.6915)	--	< 0.0001
Korea	2	1.0162 (0.6888; 1.4991)	14.6	--
Denmark	1	0.4186 (0.4025; 0.4354)	--	--
USA	2	1.0447 (0.4053; 2.6932)	99.5	--
China	1	0.2500 (0.1233; 0.5070)	--	--
Norway	1	0.3889 (0.3570; 0.4236)	--	--
Sweden	1	0.3939 (0.3745; 0.4144)	--	--
Year				
2019	1	0.5909 (0.5050; 0.6915)	--	< 0.0001
2008	1	0.8889 (0.5895; 1.3404)	--	--
2017	1	0.4186 (0.4025; 0.4354)	--	--
2021	3	0.6580 (0.3422; 1.2652)	98.6	--
2022	1	0.2500 (0.1233; 0.5070)	--	--
2015	2	0.8116 (0.1917; 3.4361)	99.8	--
Study Design				
Cohort Study	9	0.6151 (0.4159; 0.9097)	98.8	--
Intervention				
Breast-conserving surgery	3	0.5187 (0.2039; 1.3197)	86.1	0.6436
Breast-conserving surgery with RT	6	0.6625 (0.4225; 1.0389)	99.2	--
Type of Breast Cancer				
Invasive breast cancer	1	0.5909 (0.5050; 0.6915)	--	0.2678
Not specified	1	0.8889 (0.5895; 1.3404)	--	--
Early breast cancer	2	0.5192 (0.3398; 0.7933)	99.1	--
Multiple	5	0.6197 (0.2975; 1.2907)	99.3	--
Stage of Breast Cancer				
I, IIA	1	0.6471 (0.5878; 0.7123)	--	< 0.0001
I, II	2	0.4251 (0.2578; 0.7009)	94.2	--
I, II, III	4	0.3869 (0.2311; 0.6478)	98.1	--
I, IIA, IIb	1	1.6957 (1.5114; 1.9023)	--	--
I, II, III, IV	1	0.4091 (0.3490; 0.4796)	--	--
I, II	1	0.3333 (0.1141; 0.9741)	--	--

**Table 18:** Overall survival at 10 years - leave-one-out analysis The following are different influence diagnostics used in meta-analysis (specifically in R's meta for package) to detect studies that might unduly influence the overall results: rstudent: studentized residual – identifies outliers based on residuals; dffits: influence of a study on its predicted value (high values = high influence); cook.d: Cook’s distance – influence on overall model fit; cov.r: covariance ratio – influence on precision of estimates; tau2.del: between-study variance (τ²) with study deleted; QE.del: heterogeneity statistic (Q) with study deleted; hat: hat value (leverage) – influence of a study on its fitted value; Hat: duplicate of hat, possibly different formatting in table; Weight: study weight in the meta-analysis (typically inverse-variance based); is.infl: TRUE/FALSE flag indicating influential studies; Is Influential: same as is.infl.

Author	Effect	Lower	Upper	I^2^	rstudent	dffits	cook.d	cov.r	tau2.del	QE.del	Hat	Weight	Is Influential
Omitting Corradini et al. [14]	-0.7542164	-0.7802045	-0.7282282	0.989764	2.806099	0.46389427	0.215197893	1.027330	0	683.8740	0.026602493	2.6602493	No
Omitting Noh et al. [16]	-0.7506138	-0.7763041	-0.7249235	0.989746	3.013891	0.18851070	0.035536286	1.003912	0	682.6647	0.003896919	0.3896919	No
Omitting Christiansen et al. [18]	-0.6570600	-0.6909058	-0.6232143	0.988826	-8.080624	-6.96284368	48.481192128	1.742478	0	626.4517	0.426104597	42.6104597	Yes
Omitting Chu et al. [19]	-0.8001587	-0.8278672	-0.7724503	0.988286	9.704666	3.97578224	15.806844439	1.167836	0	597.5677	0.143715077	14.3715077	Yes
Omitting Li et al. [7]	-0.7473073	-0.7729644	-0.7216503	0.989835	-1.770217	-0.06423997	0.004126774	1.001317	0	688.6146	0.001315183	0.1315183	No
Omitting Hartmann-Johnsen et al. [22]	-0.7287895	-0.7556641	-0.7019148	0.989545	-4.712330	-1.47976646	2.189708768	1.098609	0	669.5422	0.089757760	8.9757760	Yes
Omitting de Boniface et al. [5]	-0.6845794	-0.7143329	-0.6548258	0.988775	-8.253908	-4.85923574	23.612171982	1.346591	0	623.6212	0.257383752	25.7383752	Yes
Omitting Van Nguyen et al [28]	-0.7497598	-0.7754194	-0.7241001	0.989733	3.157335	0.12322654	0.015184779	1.001523	0	681.7795	0.001520920	0.1520920	No
Omitting Ye et al. [29]	-0.8148975	-0.8411997	-0.7885953	0.963914	22.310741	5.10242925	26.034784256	1.052303	0	193.9791	0.049703299	4.9703299	Yes

BCS, especially with RT, was consistently associated with improved survival. The most clinically meaningful effect was seen in five-year overall survival (HR = 0.49; after outlier removal, HR = 0.42). DFS at five years also favored BCS after sensitivity analysis (HR = 0.60). These findings suggest a potential real-world advantage of BCS over MX, especially in early-stage and triple-negative breast cancer. However, wide prediction intervals and methodological differences across studies suggest that results should be interpreted cautiously.

Discussion

Breast cancer is a pervasive health concern affecting approximately one in every eight females [38]. Given its significant impact, understanding the efficacy of treatment modalities is crucial. Our systematic review and meta-analysis aim to contribute to this understanding by evaluating the outcomes of two common procedures used in treating breast cancer: MX and BCS. Our meta-analysis included six studies with 12,504 participants for local recurrence, five with 12,076 participants for regional recurrence (RR), and five with a total of 116,222 participants for mortality. Including fewer studies and a smaller sample size compared to our systematic review, which included 22 articles with a total sample size of 389,465 participants, could influence the discrepancy in results.

This meta-analysis examining local recurrence and RR rates highlights critical insights into the effectiveness of BCS compared to MX. Initially, no significant difference in local recurrence rates was found between BCS and MX (OR = 1.65, 95% CI: 0.79-3.47, p = 0.19). However, subgroup analysis by study year, as well as by breast cancer type, revealed significant variations (p = 0.01), particularly in studies published in 2019 (OR = 1.68, 95% CI: 1.27-2.22). This discrepancy can be attributed to evolving surgical techniques, advancements in adjuvant therapies, improved diagnostic methods, and updated surveillance guidelines, all of which have likely improved the precision and outcomes of BCS, reducing local recurrence rates. Subgroup analysis by breast cancer type showed significant differences, especially in invasive breast cancer, reflecting the disease's aggressiveness and varied treatment responses. Tumor biology, specifically hormone receptor status, may modify the effectiveness of surgical outcomes. Hormone receptor-positive tumors exhibit a more favorable prognosis and may respond well to adjuvant endocrine therapy, enhancing the efficacy of BCS. While triple-negative and HER2-positive subtypes tend to be more aggressive in nature and may diminish the relative benefits of BCS over MX [39]. These findings emphasize the need for tailored treatment strategies to improve clinical outcomes. In addition, shared decision-making should weigh tumor biology and treatment goals alongside quality-of-life factors. BCS often preserves body image and emotional well-being, making it a preferred option when clinically appropriate.

Our findings align with previous systematic reviews and meta-analyses, which similarly found no significant differences in local recurrence rates between BCS and MX in breast cancer patients (OR = 1.11, 95% CI: 0.61-1.99, p = 0.74) [40]. Qualitative and quantitative analyses of RR reveal significant geographic and intervention-specific variations. Korean studies showed a favorable OR of 0.39 for BCS versus MX in reducing RR, contrasting with Chinese studies reporting an OR of 4.07. Furthermore, BCS combined with RT significantly lowered OR (0.39, 95% CI: 0.22-0.70, I² = 0.0%) compared to BCS alone (OR = 4.07, 95% CI: 0.43-38.16, I² = 77.0%). High heterogeneity (I² = 83.8%) underscores variability in study methodologies and patient demographics, necessitating personalized treatment approaches. These findings substantiate existing literature advocating for BCS with RT as a preferred treatment strategy in early-stage breast cancer, supported by studies like those of Noh et al. [16] and Sang et al. [21], which underscore its efficacy in reducing local recurrence risks. Incorporating BCS with RT into treatment protocols could optimize outcomes by minimizing recurrence risks, though further research is needed to refine guidelines and enhance patient stratification.

The meta-analysis demonstrates significant benefits of BCS on overall survival at both five and 10 years. At five years, the HR is 0.49, indicating a substantial improvement in overall survival. However, high heterogeneity is observed across studies (I² = 98.8%), suggesting considerable variability in the results. Notably, differences among countries are significant, with China showing a lower HR (0.42, 95% CI: 0.17-1.06, I² = 90.5%) compared to the USA (HR = 0.69, 95% CI: 0.36-1.31, I² = 99.0%). These differences may reflect variations in healthcare systems, treatment protocols, patient demographics, genetic profiles, or lifestyle factors.

Subgroup analyses by study year revealed significant variations. For instance, studies from 2022 (HR = 0.57) might benefit from improved methodologies or larger sample sizes, reducing variability compared to earlier years. Similarly, analyses by breast cancer types showed significant differences, which could be attributed to underlying biological differences between subtypes. For example, triple-negative breast cancer (HR = 0.65) may respond differently to interventions compared to invasive breast cancer (HR = 0.56) due to distinct molecular characteristics and treatment sensitivities. Additionally, substantial variations were observed across different stages of cancer, with HRs ranging from 0.33 to 1.69, indicating varying impacts of interventions based on disease progression, prognosis, treatment intensity, and response rates.

At 10 years, the analysis focused on comparing the effectiveness of BCS with RT versus MX. Most observational studies consistently found that patients who underwent BCS with RT reported better quality of life, reduced psychological stress, and higher satisfaction rates than those who underwent MX alone. Quantitatively, the meta-analysis revealed a statistically significant reduction in the hazard of death at 10 years for patients who underwent BCS compared with MX (HR = 0.62, 95% CI: 0.42-0.91, p = 0.01). This finding aligns with the survival and quality of life benefits of BCS with RT observed in qualitative analyses.

The substantial heterogeneity (I² = 98.8%) indicates considerable variability among included studies, necessitating caution in interpretation due to the wide prediction interval of 0.15-2.60. Key studies contributing to this heterogeneity include those by Christiansen et al. [18], Chu et al. [19], and Hartmann-Johnsen et al. [22]. Variability in cancer stages, study years, selection bias, and healthcare settings are primary sources of heterogeneity. Our findings are consistent with those of De la Cruz Ku et al., who analyzed a large patient cohort and reported similar high heterogeneity (98.2%) due to patient background and cancer stages/subtypes [41]. These results highlight the need for further research to refine treatment strategies and enhance the generalizability of findings. Clinical implications are profound, as the evidence supporting BCS as a viable alternative to MX can guide clinical decision-making, offering patients less invasive options with comparable, if not better, survival outcomes. Tailoring treatments based on subtype and stage-specific characteristics could optimize survival outcomes in breast cancer patients. Continuous advancements in treatment strategies and adherence to updated clinical guidelines may further enhance overall survival rates globally. Clinicians need to use a more holistic approach, including keeping in sight the characteristics of the tumor, patient morbidities, and preferences. Considering the heterogeneity, there is a need for further research to refine treatment strategies and enhance the generalizability of these findings. 

DFS at Five Years

The qualitative analysis of the DFS at five years suggests that an improvement exists when utilizing the BCT procedure compared to MX. Despite this qualitative trend, the meta-analysis results were not significant with an HR of 0.78 under the random effects model, with a 95% CI: 0.57-1.09 and I^2^ = 98%, which indicates that there are no significant differences between therapeutic modalities. The heterogeneity levels need to be acknowledged when interpreting these results, and the possibility of publication bias given by the asymmetric result of the funnel plot results. The sources of this level of heterogeneity are possibly differences in patient demographics, tumor subtypes, cancer stages, use of RT, and follow-up durations. Previous studies have shown that these factors can significantly influence DFS outcomes. The practical utility of these results regarding the DFS at five years is that they suggest BCS with RT may offer comparable long-term control to MX, supporting its use in appropriately selected patients.

DFS at 10 Years

The analysis of DFS at 10 years revealed an HR of 1.12, indicating a 12% higher risk of disease recurrence or death in the BCS group compared to the MX group. However, this result is not statistically significant, and the analysis demonstrates substantial heterogeneity (I² = 96.4%), indicating potential variation in future study results. Subgroup analysis by country revealed significant differences (p < 0.0001), particularly for studies from Korea. These differences may be influenced by genetic factors, socioeconomic conditions, and the healthcare system. Subgroup analysis by breast cancer stage also showed significant differences (p < 0.0001) for stages I and IIA, suggesting that the stage of breast cancer might influence the comparative effectiveness of BCS and MX. These results could be explained by the sample size of the subgroup affecting the overall result. Omitting studies by Hartmann-Johnsen et al. [22] and Ye et al. [29], identified as potential outliers, resulted in an adjusted HR and reduced heterogeneity (I² = 42.3%), although the overall effect remained non-significant. The heterogeneity produced by Harmann-Johnsen et al. [22] could be due to limited demographic factors such as location (Norway), while Ye et al. contributed to the heterogeneity by specifically including females under 40, an age group where treatment outcomes can be significantly influenced. Similar to our results, a 10-year study conducted by the National Cancer Institute demonstrated no significant differences in DFS at 10 years between MX and BCS, but showed significant differences when analyzing by tumor stage [42]. A longer study conducted from 1995 to 2018 reported no significant differences as well, and no differences with subgroup analysis, providing strong support to the findings in this analysis [43].

Recent meta-analyses have consistently demonstrated that BCS offers superior survival outcomes compared to MX for early-stage breast cancer. A study using data from the National Cancer Database employed a propensity score-matched analysis to control for confounders and found improved overall and BCSS rates compared to MX [44]. Additionally, a meta-analysis with a total of 1,802,128 patients reported that patients who underwent BCS had better OS compared with MX [41]. These findings align with our results, reinforcing the notion that BCS followed by RT should be considered a viable and potentially superior option for early-stage breast cancer.

Contribution to the Literature

Our meta-analysis adds several novel insights to the existing literature. Unlike prior studies, our study offers a broader range of observational studies, representing a diverse population, enhancing the generalizability of the study. Our subgroup analyses provide an understanding of which group of patients would benefit from BCS versus MX. The incorporation of long-term data provides insight into the sustained effects of BCS and MX over an extended period, crucial for understanding the durability of treatments. By including recent data, our analysis focuses on treatments and protocols relevant to clinical practice. Comparing our analysis with previous findings reinforces our results and provides a comprehensive and updated understanding of the comparative effectiveness of BCS and MX.

Limitations

Our meta-analysis has several limitations. Initially, it relies exclusively on observational studies, which are more susceptible to bias and may lack the generalizability and validity of RCTs. The possibility of publication bias, indicated by initial asymmetry in the funnel plot, may distort the evidence pool and jeopardize the accuracy of conclusions. Additionally, wide confidence intervals and significant heterogeneity in the results indicate substantial variability in study outcomes, which should be considered when interpreting our findings. The subjective nature of assessing publication quality can lead to inconsistencies among reviewers, casting doubt on the reliability and objectivity of the study's findings. These limitations underscore the necessity for further research. Future studies should consider clinical variables, regional, and contextual factors that may impact treatment efficacy. Prioritizing research in breast cancer survival involves optimizing treatment modalities and developing tailored survivorship care models to meet the diverse long-term needs of survivors. Addressing health disparities to ensure equitable access to effective treatment is crucial for enhancing the quality of life and long-term outcomes for all breast cancer patients. Efforts to minimize study heterogeneity are essential for improving the reliability and generalizability of meta-analytic findings in this field. While our meta-analysis offers valuable insights into the impact of interventions on local recurrence, regional recurrence, and mortality in breast cancer patients, it also highlights the complexities and challenges of synthesizing data from diverse studies. Continued research and methodological advancements are necessary to address these unresolved questions and enhance our understanding of optimal treatment strategies for breast cancer.

## Conclusions

This systematic review and meta-analysis highlights the complexity of surgical decision-making in early-stage breast cancer. Across diverse populations and study designs, BCS combined with RT was consistently associated with improved overall survival and, in some subgroups, such as early-stage and triple-negative patients, better disease-free survival. These findings support the consideration of BCS plus RT as a preferred treatment strategy when anatomically and clinically feasible. Using the GRADE (grading of recommendations assessment, development and evaluation) framework, the certainty of evidence was rated as moderate for overall survival and low for recurrence outcomes, largely due to heterogeneity and the observational nature of included studies. Rather than diminishing the relevance of our findings, this underscores the need for high-quality, prospective research to better define which patients benefit most. Future studies should focus on standardizing outcomes, stratifying by molecular subtype and stage, and reducing variability in real-world practice.
